# Top-Down Effect of Direct Current Stimulation on the Nociceptive Response of Rats

**DOI:** 10.1371/journal.pone.0153506

**Published:** 2016-04-12

**Authors:** Luiz Fabio Dimov, Adriano Cardozo Franciosi, Ana Carolina Pinheiro Campos, André Russowsky Brunoni, Rosana Lima Pagano

**Affiliations:** 1 Laboratory of Neuromodulation and Experimental Pain, Hospital Sírio Libanês, Rua Prof Daher Cutait, 69, Sao Paulo, SP, 01308–060, Brazil; 2 Service of Interdisciplinary Neuromodulation (SIN), Department and Institute of Psychiatry, Faculty of Medicine of University of São Paulo, Laboratory of Neuroscience (LIM27), Department and Institute of Psychiatry, University of São Paulo, Rua Doutor Ovidio Pires de Campos, 785, Sao Paulo, SP, 05403–000, Brazil; 3 Center for Clinical and Epidemiological Research & Interdisciplinary Center for Applied Neuromodulation (CINA), University Hospital, University of São Paulo, São Paulo, Avenida Professor Lineu Prestes 2565, ext. 3, Sao Paulo, SP, 05508–000, Brazil; University Medical Center Goettingen, GERMANY

## Abstract

Transcranial direct current stimulation (tDCS) is an emerging, noninvasive technique of neurostimulation for treating pain. However, the mechanisms and pathways involved in its analgesic effects are poorly understood. Therefore, we investigated the effects of direct current stimulation (DCS) on thermal and mechanical nociceptive thresholds and on the activation of the midbrain periaqueductal gray (PAG) and the dorsal horn of the spinal cord (DHSC) in rats; these central nervous system areas are associated with pain processing. Male Wistar rats underwent cathodal DCS of the motor cortex and, while still under stimulation, were evaluated using tail-flick and paw pressure nociceptive tests. Sham stimulation and naive rats were used as controls. We used a randomized design; the assays were not blinded to the experimenter. Immunoreactivity of the early growth response gene 1 (Egr-1), which is a marker of neuronal activation, was evaluated in the PAG and DHSC, and enkephalin immunoreactivity was evaluated in the DHSC. DCS did not change the thermal nociceptive threshold; however, it increased the mechanical nociceptive threshold of both hind paws compared with that of controls, characterizing a topographical effect. DCS decreased the Egr-1 labeling in the PAG and DHSC as well as the immunoreactivity of spinal enkephalin. Altogether, the data suggest that DCS disinhibits the midbrain descending analgesic pathway, consequently inhibiting spinal nociceptive neurons and causing an increase in the nociceptive threshold. This study reinforces the idea that the motor cortex participates in the neurocircuitry that is involved in analgesia and further clarifies the mechanisms of action of tDCS in pain treatment.

## Introduction

Transcranial direct current stimulation (tDCS) is a noninvasive brain stimulation technique with promising clinical outcomes for various conditions, including Parkinson’s disease [1], stroke [2], multiple sclerosis [3], schizophrenia [4], major depression [5] and pain [6]. This approach is relatively safe, well tolerated, affordable and easily deployable and consists of the application of a weak electric current to the scalp via two electrodes (anode and cathode); its efficacy depends critically on parameters such as electrode position and current strength [7]. The clinical effects of tDCS have been attributed to modulation of neuronal plasticity and synaptic connections [8–11], an increase in cortical excitability beyond the stimulation period [7,12] and the consolidation of this neuroplasticity, which is regulated by several mechanisms including acetylcholine, dopamine, serotonin, GABA, Na+ channel and NMDA-receptor activity [8,13–17]. Additional studies have shown a prolonged facilitation or inhibition of subcortical neuron activation, both in humans and in animals [18,19].

The analgesia induced by tDCS when applied over the primary motor cortex (M1) has been attributed to modulation of areas associated with pain processing, including the anterior cingulate, insula, thalamic nuclei and upper brainstem [9,20–25], as well as to regulation of glutamate, GABA and opioid activity, which results in the activation of descending analgesic pathways [25–28]. Evidence suggests that anodal tDCS increases the excitability of the underlying motor cortex, whereas cathodal tDCS diminishes cortical excitability [12,29,30]. Anodal tDCS over M1 produces long-lasting therapeutic effects in chronic pain conditions, including fibromyalgia [31–33], central pain [34,35], and phantom limb pain [36]. Experimental anodal direct current stimulation (DCS) in rats has been shown to induce thermal antinociceptive effects [37] and to reverse inflammatory chronic pain [38] and chronic stress-induced pain via the inhibition of hippocampal TNFα and spinal BDNF levels [39,40]. In addition, anodal DCS has been found to reverse mechanical hyperalgesia and the behavioral alterations in neuropathic rats, which is accompanied by changes in the BDNF and cytokine levels in different areas of the central nervous system [41,42]. Cathodal tDCS over M1 also induces analgesia in patients with chronic pain [43,44]. Both anodal and cathodal tDCS are known to increase the nociceptive threshold in healthy subjects [22,44–47]; however, until now, no experimental studies have been conducted to evaluate the cathodal DCS-induced antinociception in naive rats to better understand the neurocircuitry that mediates this response.

The expression of inducible transcription factors (ITFs) or immediate early genes, including *egr-1* (*zif-268*, *krox-24*, or *zenk*), *c*-fos and *c-jun*, has been widely used to map neuronal activation under different physiological and non-physiological conditions [48–50]. ITFs are induced in neurons in response to extracellular stimuli, including depolarization, growth factors and neurotransmitters such as glutamate and substance P [51–56]. ITF expression can be elicited by various types of noxious stimuli, including chemical, thermal and mechanical stimuli, in different areas of the brain and the spinal cord. These factors thus become important tools for the study of the neural circuitry underlying nociception [50,57–61]. A direct correlation between decrease in ITF expression and antinociception in the DHSC has been suggested [62–64]. Few studies have evaluated the relationship between cortical stimulation, analgesia and ITFs. Using Egr-1 and c-Fos immunoreactivity (IR), epidural motor cortex stimulation has been shown to induce analgesia in naive and neuropathic rats, inhibiting the spinal neurons and activating the PAG [65–67]. Although a top-down effect of tDCS has been hypothesized, which would lead to activation of the descending analgesic pathway and consequently an increase in the nociceptive threshold, its inhibitory action on spinal nociceptive neurons has yet to be demonstrated.

The present study was conducted to investigate the effects of cathodal DCS on the thermal and mechanical pain sensitivity of healthy rats and to evaluate the pattern of neuronal activation using Egr-1 detection in the midbrain periaqueductal gray (PAG), which is a key component of the descending analgesic pathway, and in the dorsal horn of the spinal cord (DHSC), which is the region where nociceptive information is received, integrated, and relayed to higher brain areas. In addition, to evaluate the involvement of the spinal opioid system, enkephalin labeling in the DHSC was also investigated.

## Materials and Methods

### Experimental design

**Fig 1:**
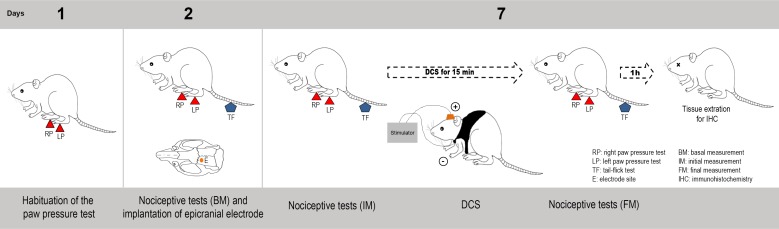
Experimental design. Rats were habituated to the paw pressure test in the day 1. In the second day, they were evaluated in the paw pressure and tail flick tests, and subsequently, under anesthesia, epicranial electrodes were implanted over their left motor cortex. After 5 days (day 7), the nociceptive tests were performed again. A group of rats received a single session of 15 minutes of DCS (250 μA), after which, they were re-evaluated on the nociceptive tests while still under stimulation. Rats that were submitted to the surgical procedures but were not electrically stimulated (sham) and rats that did not receive any surgical procedure (naive) were also evaluated. After 1 hour of the last nociceptive evaluation, the animals were anesthetized, perfused, and their tissue fragments were processed for immunohistochemical analysis.

Adult rats were evaluated with nociceptive tests (described in the *Measuring nociceptive threshold* section below), and subsequently, under anesthesia, epicranial electrodes were implanted over their left motor cortex involving the functional area of the right hind limb. After 5 days, the nociceptive tests were performed again in the animals while they were awake. Next, a group of rats received a single session of 15 minutes of DCS, after which, they were re-evaluated on the nociceptive tests while still under stimulation. Rats that were submitted to the surgical procedures but were not electrically stimulated (sham) and rats that did not receive any surgical procedure (naive) were also evaluated. After 1 hour of the last nociceptive evaluation, the animals were anesthetized, perfused, and their tissue fragments were processed for immunohistochemical analysis to evaluate the neuronal activation pattern in the PAG and DHSC and enkephalin expression in the DHSC (Fig 1).

### Animals

Twenty-five male Wistar rats (180–220 g) were housed in acrylic boxes (three rats per cage) for at least two days before the initiation of the experimental procedures. The boxes were kept at a constant ambient room temperature with a controlled temperature (22°±2°C) and a light/dark cycle (12/12 h), and they had wood shavings and free access to water and rat chow pellets. All procedures were in accordance with the guidelines on the ethical use of animals in research involving pain and nociception [68] and were approved by the Ethics Committee on the Use of Animals at Hospital Sírio Libanês (Sao Paulo, Brazil) under protocol number CEUA 2011/24.

### Electrode implantation and electrical stimulation

Mimicking the clinical protocol that induces analgesia in healthy humans, we decided to use cathodal DCS on naive rats in our study. The electrode fixation and the stimulation protocol were applied according to an earlier study [69]. Briefly, rats were deeply anesthetized with ketamine/xylazine (50/20 mg/kg, intraperitoneal) as well as with a local scalp injection of 2% lidocaine (100 μL/animal, subcutaneous). Then, under stereotactic guidance using a map developed by our group [70], an epicranial electrode that consisted of a tubular plastic jacket was fixed onto the skull over the left primary motor cortex (M1) in the functional area of the right hind limb (1 mm caudal to bregma; 1 mm left to midline). Rats were treated with ketoprofen (10 mg/kg, subcutaneous) for three days after surgery. Five days after implantation of the electrode, the epicranial jacket was filled with a conductive gel (Electron Plus^®^, Hal, SP, Brazil), and the cathode was inserted. The animals were dressed in vests that placed a large rubber plate on the ventral thorax, which served as the counter electrode (anode). The electrodes were connected to an electrical stimulator (Striat^®^, IBRAMED, SP, Brazil) with wire leads, which allowed the animals to move freely in the cage (S1 Fig). The experimental group received a single DCS session of 15 minutes (250 μA over an area of 2.27 mm^2^), and the final measurements during the nociceptive tests were recorded while the rats were still under stimulation. The sham group was subjected to the same conditions but did not receive stimulation. The rats were randomly divided into the sham and stimulated groups. In total, the animals were divided into three groups: rats with no surgical procedure (naive, n = 7), rats with an epicranial electrode and false stimulation (sham, n = 7), and stimulated rats (n = 7). Four animals were excluded from the study because they removed their implants before the final nociceptive tests.

### Measuring nociceptive threshold

Nociceptive tests were conducted prior to the electrode implantation and five days after, before (initial measurement) and during DCS (final measurement). Naive and sham rats were also evaluated. On the day of the test, the animals were brought into a separate quiet room 1 hour before the nociceptive tests to allow them to habituate to the environment. The thermal nociceptive threshold was determined using the tail-flick test, which has been previously described [71]. Briefly, radiant heat was focused on the lower third of their tail using an analgesiometer (EEF 300, Insight^®^, SP, Brazil). Movement of the tail activated a photocell, thereby turning off both the light and a reaction timer. The nociceptive threshold was defined as the time necessary to induce the tail-flick response. The mechanical nociceptive threshold was determined using a pressure apparatus on the right hind paw (EEF-440, Insight^®^), which has also been previously described [72]. Briefly, the mass (in grams) required to induce a withdrawal response represented the nociceptive threshold. The results were analyzed by comparing between the initial and final measurements. In order to reduce animal stress, the rats were handled by only one experimenter and were habituated to the paw pressure test the day preceding the electrode implantation. Both nociceptive tests were performed before electrode implantation (basal measurement) to evaluate if the nociceptive threshold could be changed after surgical procedure.

### Immunohistochemistry

One hour after the last nociceptive test, the rats were deeply anesthetized with ketamine and xylazine and then subjected to transcardiac perfusion with saline solution followed by 4% paraformaldehyde (PFA) dissolved in 0.1 M phosphate buffer (PB). The animals were perfused 1 hour after the last nociceptive stimulus because the peak expressions of ITF proteins occur approximately 1 hour after the stimulus and fade by 3 to 4 hours post-stimulation [50]. The brain and lumbar spinal cord (L2-L5 segments) were collected and post-fixed in PFA for 4 hours, followed by incubation with 30% sucrose solution in PB for 48 hours at 4°C. Tissue sections (30 μm) were cut on a freezing microtome, washed in PB, and incubated for 12 to 16 hours at 4°C with rabbit anti-Egr-1 (Zif268, 1:1000, C-19; Santa Cruz Biotechnology^®^, CA, USA) or mouse anti-enkephalin (ENK, 1:1000, MAB350, Millipore^®,^ CA, USA) primary antibodies that were diluted in 0.3% of Triton X-100 containing 5% normal donkey serum (Jackson ImmunoResearch^®^, ME, USA). Then, sections were incubated for 2 hours at room temperature with biotinylated secondary antibodies (Jackson ImmunoResearch^®^, ME, USA) and incubated with an avidin-biotin complex (1:100, ABC Elite kit, Vector Labs^®^, CA, USA) for 2 hours at room temperature. The sections were visualized with 0.05% diaminobenzidine tetrahydrochloride (DAB, Sigma-Aldrich^®^, MO, USA) and 0.03% (final concentration) hydrogen peroxide in PB. The sections were then mounted on glass slides, air-dried, dehydrated, and coverslipped with Permount (Fisher Scientific^®^, PA, USA). The samples were washed between each step (3 x 10 min). The immunoreactivity was analyzed using a light microscope (E1000, Nikon^®^, NY, USA) and ImageJ software (National Institute of Health, MD, USA; http://rsbweb.nih.gov/ij/). Figures were assembled using Adobe Photoshop (Adobe Systems, CA, USA); the images were converted to black and white and optimized using contrast and brightness only. Quantitative analysis was performed to determine the density of nuclei showing positive immunoreactivity (IR) for Egr-1 in the rostral portion of the PAG (dorsomedial, dorsolateral, lateral and ventrolateral columns) and DHSC (laminae I-IV) and the IR of ENK in the DHSC (laminae I-VI). Measurements were taken from 7 different sections for each analyzed animal. The regions of interest were identified based on a stereotaxic atlas [73] and an atlas of the spinal cord [74].

### Statistical analysis

Data are presented as the mean ± standard error of the mean. Statistical analyses were conducted with GraphPad Prism^®^ 5.0 software (GraphPad Software Inc; La Jolla, CA, USA). The results of the nociceptive tests were analyzed using two-way repeated measures analysis of variance (ANOVA) followed by the Bonferroni post hoc test. Immunohistochemistry data were analyzed using one-way ANOVA followed by the Bonferroni post hoc test. In all cases, *p* < 0.05 was considered statistically significant.

## Results

**Fig 2:**
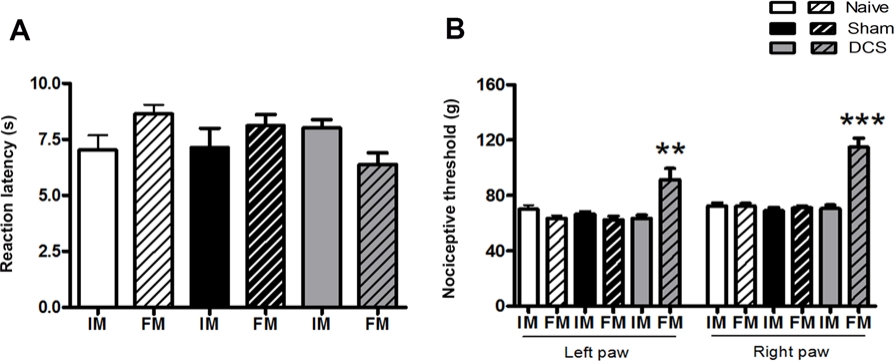
DCS and nociceptive response. Nociceptive threshold was evaluated using the tail-flick test (A) and paw pressure test (B) before (initial measurement, IM) and during DCS (final measurement, FM) in naive (without surgical intervention), sham (with epicranial electrode without stimulation) and stimulated (DCS, 250 μA, 15 min) rats. Epicranial electrodes were placed in the left hemisphere. Values represent the mean ± SEM (n = 7 animals per group). *p < 0.05, when compared to the naive group. DCS: direct current stimulation.

Cathodal DCS did not change the thermal nociceptive threshold in the tail of the animals (Factor Time x Factor Treatment, F_(2,9)_ = 0.08030, *p* = 0.92) (Fig 2A). However, it was able to increase the mechanical nociceptive threshold of the left (Factor Time x Factor Treatment, F_(2,27)_ = 15.37, *p* < 0.0001) and right (Factor Time x Factor Treatment, F_(2,27)_ = 32.60, *p* < 0.0001) hind paws (53% and 73% increases, respectively) compared with that of the naive and sham animals (Fig 2B). The results obtained in the nociceptive tests, before electrode implantation in the basal measurement (data not showed), were equal to the data observed after 5 days of the surgical procedure, showing that the increase of nociceptive threshold after the DCS was a consequence of the electrical stimulation.

**Fig 3:**
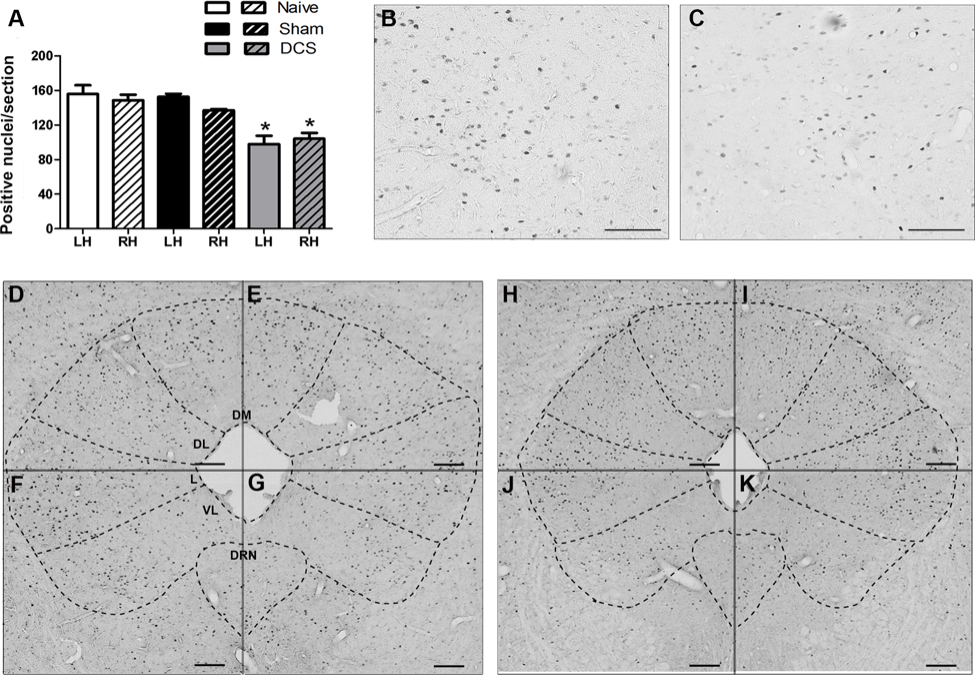
DCS and neuronal activation in the PAG. Quantification of the Egr-1-positive nuclei in the PAG of naive (without surgical intervention), sham (with epicranial electrode without stimulation) and stimulated (DCS, 250 μA, 15 min) rats. Values represent the mean ± SEM (n = 7 animals per group). *p < 0.05, when compared to the naive group (A). Photomicrographs show the Egr-1-positive nuclei staining in the PAG of naive (B, D-G) and stimulated (C, H-K) rats. Figs B and C represent higher magnification insets of the ventrolateral PAG of naive and stimulated rats, respectively. Sections of the left (B-D, F, H, J) and right (E, G, I, K) hemisphere of the PAG. Scale bars: 50 μm (B and C) and 100 μm (D-K). DCS: direct current stimulation; DL: dorsolateral PAG; DM: dorsomedial PAG; DRN: dorsal raphe nucleus; L: lateral PAG; LH: left hemisphere; PAG: midbrain periaqueductal gray; RH: right hemisphere; VL: ventrolateral PAG.

**Fig 4:**
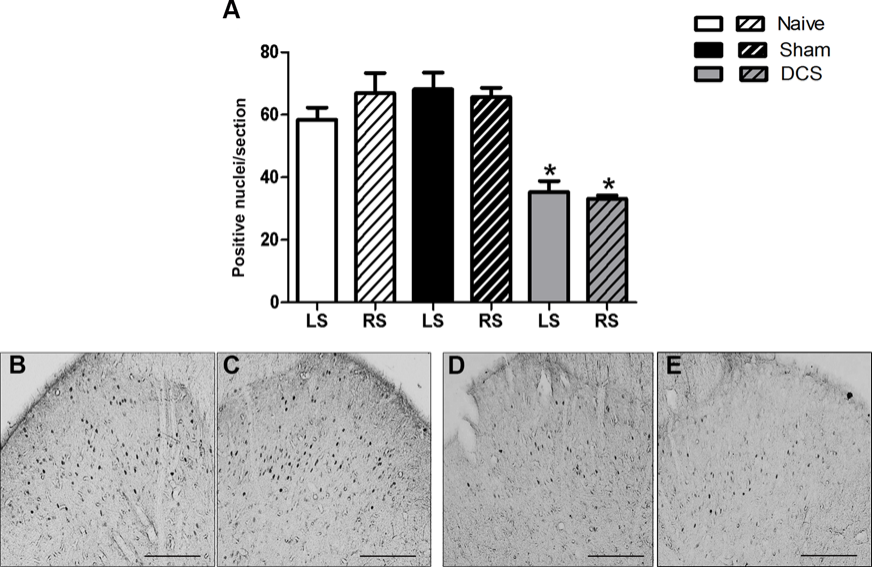
DCS and neuronal activation in the DHSC. Quantification of the Egr-1-positive nuclei in the DHSC of naive (without surgical intervention), sham (with epicranial electrode without stimulation) and stimulated (DCS, 250 μA, 15 min) rats. Values represent the mean ± SEM (n = 7 animals per group). *p < 0.05, when compared to the naive group (A). Photomicrographs show the Egr-1-positive nuclei staining in the superficial laminae of the DHSC of naive (B, C) and stimulated (D, E) rats. Sections of the left (B, D) and right (C, E) DHSC. Scale bars: 50 μm. DCS: direct current stimulation; DHSC: dorsal horn of the spinal cord; LS: left side; RS: right side.

The immunohistochemistry analysis showed a bilateral decrease in Egr-1-positive neurons in the PAG after DCS (F_(2,11)_ = 12.10, *p* = 0.0028, left hemisphere; F_(2,12)_ = 15.30, *p* = 0.0009, right hemisphere), primarily in the ventrolateral PAG (Fig 3), compared with the results of the control groups. Furthermore, our results showed that DCS bilaterally decreased (F_(2,9)_ = 17.05, *p* = 0.0020, left side; F_(2,9)_ = 28.93, *p* = 0.0004, right side) the Egr-1-positive neurons in the DHSC (Fig 4) when compared with control animals.

**Fig 5:**
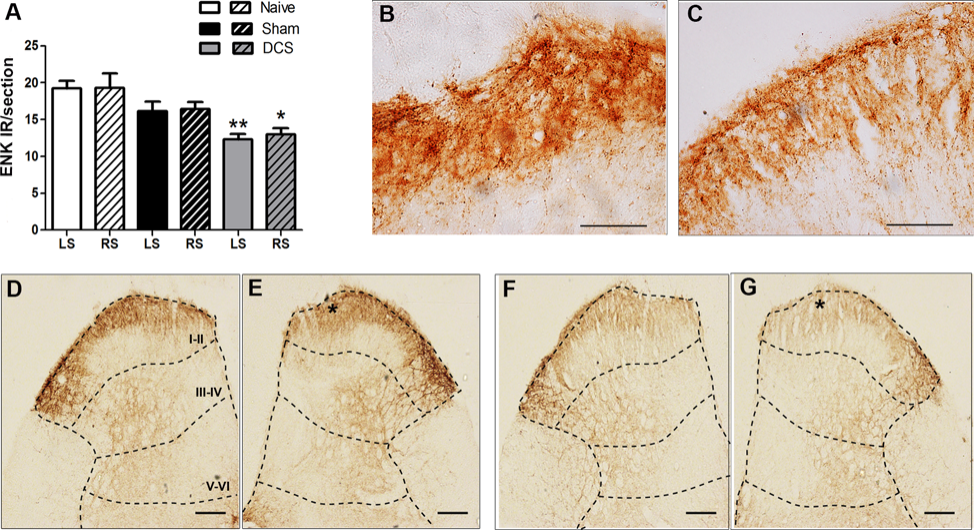
DCS and enkephalin immunostaining in the DHSC. Quantification of the enkephalin (ENK) immunoreactivity (IR) in the DHSC of naive (without surgical intervention), sham (with epicranial electrode without stimulation) and stimulated (DCS, 250 μA, 15 min) rats. Values represent the mean ± SEM (n = 7 animals per group). *p < 0.05, when compared to the naive group (A). Photomicrographs show the ENK-IR in the DHSC of naive (B, D, E) and stimulated (C, F, G) rats. Figs B and C show higher magnification insets of laminae I-II of naive and stimulated animals, indicated by asterisks in figs E and G, respectively. Sections of the left (D, F) and right (B, C, E, G) DHSC. Scale bars: 50 μm (B and C) and 100 μm (D-G). Laminae I-VI of the DHSC are represented in figs D-G. DCS: direct current stimulation; DHSC: dorsal horn of the spinal cord; LS: left side; RS: right side.

Cathodal DCS bilaterally decreased (F_(2,11)_ = 11.06, *p* = 0.0038, left side; F_(2,11)_ = 5.503, *p* = 0.0275, right side) the spinal ENK immunoreactivity compared with that of the naive group (Fig 5).

## Discussion

tDCS has been used in the treatment of various chronic pain disorders [31–36]; however, its mechanism of action remains poorly understood. We showed here that cathodal DCS, applied over the functional area of the hind limb, did not interfere with the thermal nociceptive threshold in the tail. Evidence indicates that the analgesic effect induced by cortical stimulation depends on the electrode position in the cortical somatotopy representation of pain perception [75,76]. In this regard, antinociception induced by epidural motor cortex stimulation in healthy rats was shown to have a topographic relationship with the stimulated area of the M1 [67]. Our results confirm this somatotopic analgesic effect induced by cortical stimulation, as cathodal DCS did not alter the nociceptive threshold in the tail but induced a bilateral increase of the mechanical nociceptive threshold in the hind paws of rats. In contrast with our findings, anodal DCS over the M1 has been shown to increase the nociceptive threshold in naive rats in the tail-flick test [37]. However, our results are consistent with findings obtained in healthy humans who were subjected to cathodal tDCS of the motor cortex; this procedure resulted in an increase in the cold and mechanical detection thresholds but not in the heat threshold, suggesting that cathodal tDCS temporarily reduces sensitivity to the A-fibers mediating the somatosensory inputs [46]. Interestingly, similar to the bilaterality that was observed in our results for the mechanical threshold, the aforementioned study also demonstrated bilateral changes in the cold detection threshold [46].

Another difference that could account for the differential antinociception observed in this study is the polarity-specific alteration in cortical blood flow (CBF) induced by tDCS. Cathodal tDCS results in a decrease in CBF in the cortical area, whereas anodal tDCS results in an increase [77]. In addition to this localized effect, the CBF decrease induced by cathodal tDCS spreads to target-adjacent areas [78]. This widespread modulation could, in turn, account for the widespread effect (namely, the bilateral antinociception) that was observed in our results. Additionally, another hypothesis considers diffuse noxious inhibitory control (DNIC) activation, which refers to the supraspinally mediated inhibitory process that occurs in the pain-signaling neurons of the DHSC in response to heterogenic noxious stimuli [79, 80]. In fact, tDCS displays synergistic effects when combined with a DNIC approach [22]. As suggested, a plausible mechanism could be that tDCS elicits activation of the same neural circuits that are associated with DNIC, particularly the endogenous descending analgesic pathways [22].

The most prominent neurocircuitry involved in the descending control of pain is the PAG, as activating the rostral ventromedial medulla and the locus ceruleus, which project their serotonergic and noradrenergic descending fibers, respectively, to the DHSC, results in the specific inhibition of the firing of spinal nociceptive neurons [81–83]. To evaluate whether the cathodal DCS-induced antinociception in naive rats was mediated by activation of the descending analgesic system, we evaluated the neuronal activation in the PAG and DHSC. DCS bilaterally decreased the Egr-1 immunolabeling in the PAG, primarily in the ventrolateral and lateral subdivision, and in the superficial laminae of the DHSC. Based on anatomic and functional patterns, the PAG has been subdivided into four columns: the dorsomedial, dorsolateral, lateral and ventrolateral [84]. Antinociception effects of different types are mediated by the lateral and ventrolateral PAG [85–87]; our findings corroborate this idea, as the changes in the activation pattern that occurred in these subdivisions were more evident. As previously shown, epidural motor cortex stimulation induces antinociception in healthy rats by enhancing the neuronal firing rate and Fos immunoreactivity within the PAG and, by simultaneously decreasing GABA and Egr-1 labeling; this suggests an inhibition of local GABAergic interneurons with subsequent activation of the excitatory neurons responsible for descending analgesic control [66]. Similarly, França et al. [67] also showed an increase in Fos immunolabeling in the different subdivisions of the PAG of naive rats after epidural stimulation; however, this increase was also observed in Egr-1. Further investigation of the PAG neurocircuitry involved in the tDCS-induced analgesic effect is needed to clarify which populations of neurons are modulated by this therapeutic intervention. Our results also suggest that cathodal DCS induces a disinhibition of the PAG with resulting activation of the descending analgesic pathways, increasing the nociceptive threshold through a top-down modulation mechanism, as previous proposed [22,26,40]. The immunohistochemistry results observed in this study demonstrate that the results of behavioral studies are consistent with both altered activation of the PAG, which is the main region of the midbrain descending analgesic pathway, and a decrease in the activation of spinal nociceptive neurons, which are the main input for ascending nociceptive information. As the expression of Egr-1 in the DHSC is directly related to the activity of nociceptive neurons [59], our results suggest that DCS leads to a decrease in the transmission of ascending nociceptive information. These results are supported by others who have demonstrated, after epidural cortical stimulation, a relationship between analgesia and decreased spinal Egr-1 in naive and neuropathic rats [65,67].

A growing body of evidence has indicated the participation of the opioid system in the analgesia induced by cortical stimulation [27,28,88–91]. In an attempt to evaluate the involvement of the spinal opioid system in the antinociception induced by cathodal DCS in healthy rats, we evaluated enkephalin (ENK) labeling in the DHSC. Enkephalins are endogenous opiates that play an important role in the modulation of nociceptive information by mediating synaptic transmission in several areas of the central nervous system [92,93]. Most ENK present in the DHSC originates from local interneurons, although a few enkephalinergic fibers may descend from the brainstem [94–96] or originate from dorsal root ganglion neurons [97, 98]. In the DHSC, ENK is highly concentrated in the neurons located in the superficial laminae I and II and is sparsely distributed in the deep laminae V–VII and around the central canal [99, 100]. Additionally, ENK binds to both μ and δ opioid receptors with similar affinities but is inactive at κ opioid receptors [101]. Spinal ENK inhibits neurotransmitter release by the nociceptive primary afferents and the spinothalamic projection neurons in response to noxious peripheral stimuli [102–104]. Indeed, a positive correlation between an increase in spinal ENK and analgesia in chronic pain conditions has been shown [105–107]. Nevertheless, in the absence of persistent pain, spinal opioid release has been shown to be inhibited by activation of the descending analgesic system [108,109], which could serve to shut down the spinal opioid system whenever the descending serotonergic and noradrenergic pathways are active, thus preventing undesirable analgesia under normal conditions. Our findings are consistent with this process; our results show that during the DCS-induced antinociception in healthy rats, a decrease in spinal ENK concomitant with an inhibition of nociceptive neurons in the DHSC occurs, likely due to activation of the descending analgesic system.

The brainstem serotonergic and noradrenergic fibers are the main components of the descending analgesic pathways, as the release of their biogenic amines in the DHSC result in inhibition of spinal nociceptive neurons [82]. However, although the majority of serotonergic fibers act directly on the spinal nociceptive neurons, the noradrenergic fibers have been shown to act indirectly through the secondary release of opiate-like substances [110,111]. As DCS is a modulatory intervention that targets the endogenous neural circuits, it could induce a differential activation of such parallel pathways, resulting in enhanced serotonergic fiber-mediated and reduced noradrenergic fiber-mediated effects and a subsequent decrease in ENK release. Despite this differential modulation, the net effect is still the activation of the descending analgesic system culminating with inhibition of the spinal nociceptive neurons. This hypothesis also explains our results, which showed a decrease in both ENK and Egr-1 immunoreactivity in the DHSC.

Altogether, the present findings suggest that DCS induces disinhibition of the midbrain descending analgesic pathway with a subsequent inhibition of spinal nociceptive neurons, leading to an increase in the nociceptive threshold. This study reinforces the idea that the motor cortex is involved in the neural circuits that control the nociceptive response and highlights the importance of further studies evaluating the clinical use of tDCS as an alternative for pain control.

## Supporting Information

S1 Fig Cathodal DCS treatment. The epicranial electrode was fixed onto the skull over the left primary motor cortex (1 mm caudal to bregma; 1 mm left to midline). Before DCS, the epicranial jacket was filled with a conductive gel and the cathode was inserted. The animals were dressed in vests that placed a large rubber plate on the ventral thorax, which served as the counter electrode (anode). The electrodes were connected to an electrical stimulator with wire leads, which allowed the animals to move freely in the cage. Afterward, rats received a single DCS session of 15 minutes.
(TIF)

## Abbreviations

DCS: direct current stimulation
DHSC: dorsal horn of the spinal cord
M1: primary motor cortex
PAG: midbrain periaqueductal gray
tDCS: transcranial direct current stimulation
