# Supplementary figures and images for: Top-Down Effect of Direct Current Stimulation on the Nociceptive Response of Rats
Source: PLoS One. 2016 Apr 12;11(4):e0153506. doi: 10.1371/journal.pone.0153506 (PMC4829148; doi:10.1371/journal.pone.0153506)

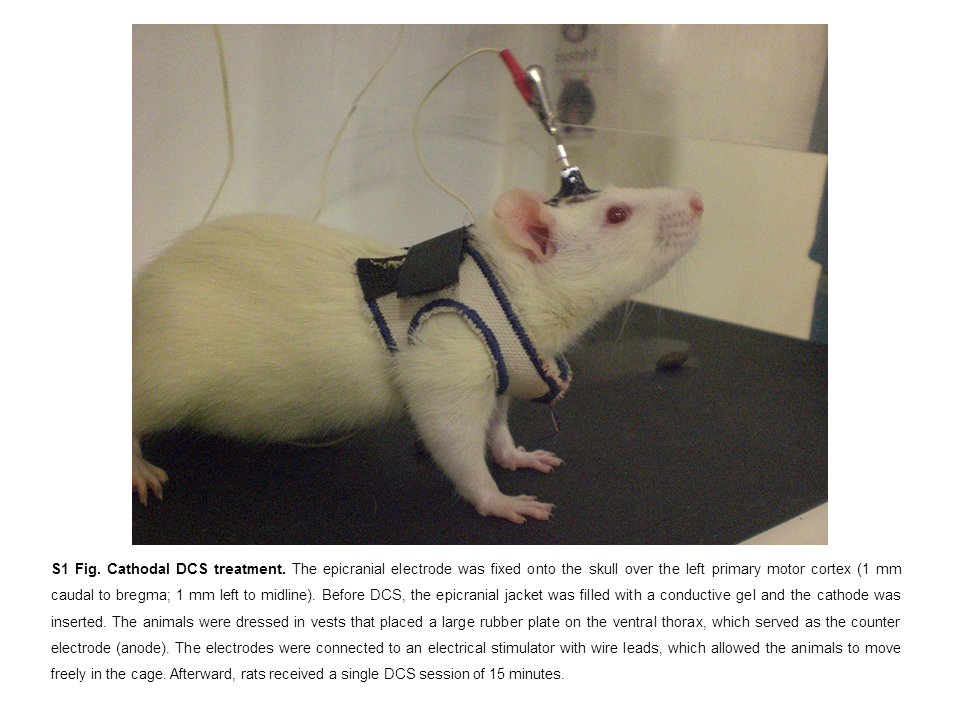

Supplement: S1 Fig — The epicranial electrode was fixed onto the skull over the left primary motor cortex (1 mm caudal to bregma; 1 mm left to midline). Before DCS, the epicranial jacket was filled with a conductive gel and the cathode was inserted. The animals were dressed in vests that placed a large rubber plate on the ventral thorax, which served as the counter electrode (anode). The electrodes were connected to an electrical stimulator with wire leads, which allowed the animals to move freely in the cage. Afterward, rats received a single DCS session of 15 minutes. (TIF) [file pone.0153506.s001.tif]
